# Corneal Endothelial Cell Loss in Shallow Anterior Chamber Eyes After Phacoemulsification Using the Eight-Chop Technique

**DOI:** 10.3390/jcm14093045

**Published:** 2025-04-28

**Authors:** Tsuyoshi Sato

**Affiliations:** Department of Ophthalmology, Sato Eye Clinic, Nemoto 3-3, Matsudo-shi 271-0077, Chiba-ken, Japan; perfect-eightchop@sato-ganka.com

**Keywords:** cataract surgery, corneal endothelial cell, eight-chop technique, shallow anterior chamber, phacoemulsification

## Abstract

**Objectives:** In this study, the correlation between anterior chamber depth (ACD) and corneal endothelial cell density (CECD) loss was evaluated, and an assessment was made of the safety and efficacy of the eight-chop technique in cataract surgery for patients with shallow anterior chamber (SAC) depth. **Methods:** The technique was applied to patients with SAC and normal ACD, defined as <3 mm and ≥3 mm, respectively. Best-corrected visual acuity (BCVA), intraocular pressure (IOP), CECD, coefficient of variation, percentage of hexagonal cells, and central corneal thickness were assessed pre- and postoperatively. Operative time, phaco time, aspiration time, cumulative dissipated energy (CDE), and volume of fluid used were recorded intraoperatively. **Results:** A total of 180 eyes from 99 patients (mean age, 74.8 ± 5.1 years; 28 men, 71 women) were analyzed. In the SAC group, the mean operative time, phaco time, aspiration time, CDE, and volume of fluid used were 4.7 min, 15.4 s, 65.6 s, 5.87, and 26.6 mL, respectively, demonstrating favorable surgical metrics. CECD loss was 1.3% at 7 weeks, 1.1% at 19 weeks, and 0.9% at 1 year, with no significant decrease after surgery in the SAC group. No correlation was observed between CECD loss and ACD in either group. **Conclusions:** These findings suggest that the eight-chop technique is a minimally invasive and effective approach that preserves corneal endothelial integrity, even in patients with SAC depth.

## 1. Introduction

Cataract surgery is the most commonly performed ophthalmic procedure worldwide [1,2], offering significant improvements in visual function and serving as a phacoemulsification technology. Nevertheless, in accordance with the risks associated with any surgical procedure, there is a possibility of postoperative complications, even when utilizing the most advanced phacoemulsification technology [3]. During the surgery, corneal endothelial cells are particularly important owing to their non-regenerative nature. Unlike other ocular cells, corneal endothelial cells do not proliferate; thus, when endothelial cells are lost, compensation occurs through cell migration, enlargement, and an increase in heterogeneity [4]. Corneal endothelial cell density (CECD) serves as a key biomarker for assessing surgical trauma to the cornea. A critically low CECD, particularly below 400 cells/mm^2^, leads to endothelial decompensation, resulting in impaired corneal detumescence and subsequent overhydration [5,6,7]. This process manifests clinically as corneal edema, stromal thickening, bullous keratopathy, and corneal scarring, ultimately leading to irreversible visual acuity loss [5,6,7]. Pseudophakic bullous keratopathy remains a significant complication, with an incidence of approximately 1% to 2% [8].

The rate of CECD loss following phacoemulsification has been attributed to many factors [9,10,11], including direct instrument trauma, exposure to ultrasonic energy, oxidative stress induced by free radical formation, and mechanical contact with intraocular lenses or lens nuclear fragments [12,13]. Thus, it is essential to assess the preoperative and intraoperative factors contributing to CECD loss to optimize surgical outcomes.

To comprehensively evaluate these risks, it is necessary to investigate the association between patient age, nuclear density, operative time, phaco time, cumulative dissipated energy (CDE), aspiration time, and volume of fluid used with CECD loss. These intraoperative variables play a crucial role in determining the extent of endothelial trauma.

Furthermore, since phacoemulsification is performed within a confined intraocular space, ensuring adequate surgical working depth can mitigate the risk of endothelial damage. A deeper anterior chamber may provide additional protection against mechanical and thermal insult during surgery. However, conflicting evidence exists regarding the role of anterior chamber depth (ACD) in CECD loss, as some studies have reported [14,15]. Studies have systematically analyzed the relationship between intraoperative parameters—including operative time, phaco time, CDE, aspiration time, and volume of fluid used—and CECD loss. Nonetheless, further investigation is necessary to clarify these relationships and optimize surgical techniques to preserve endothelial integrity.

Cataract surgery utilizing the eight-chop technique has been reported to require less operative time, phaco time, CDE, aspiration time, and volume of fluid used compared with other phacoemulsification techniques [16,17]. By performing cataract surgery using the eight-chop technique in patients with varying ACDs and analyzing intraoperative parameters along with postoperative CECD loss, this study aims to assess the efficacy of the eight-chop technique in patients with shallow anterior chamber (SAC) depth. Additionally, as early studies on patients with SAC depth no longer reflect current surgical outcomes because of advancements in phacoemulsification technology, this research seeks to clarify the relationship between CECD loss and ACD using state-of-the-art phacoemulsification systems.

The primary objective of this study was to evaluate intraoperative parameters and postoperative corneal endothelial cell changes in patients with cataracts of different ACDs who underwent surgery with the eight-chop technique. This study further aimed to investigate the correlation between ACD and CECD loss and assess the overall efficacy of the eight-chop technique in patients with SAC depth.

## 2. Materials and Methods

### 2.1. Study Population

Patients who underwent phacoemulsification and posterior chamber intraocular lens (IOL) implantation between June 2022 and November 2023 at our clinic were included in this study. Patients were categorized based on preoperative ACD, with those having ACD ≥ 3.0 mm classified as the control group and those with ACD < 3.0 mm assigned to the SAC group [18,19]. Patients were excluded if they had corneal disease or opacity, uveitis, or a history of trauma or surgery.

### 2.2. Preoperative Assessment

All patients underwent a comprehensive ophthalmic evaluation prior to surgery, including slit-lamp biomicroscopy and retinal assessment. Best-corrected visual acuity (BCVA) and intraocular pressure (IOP) were documented as part of the standard preoperative assessment. Corneal endothelial parameters—including CECD (cells/mm^2^), central corneal thickness (CCT), coefficient of variation (CV), and percentage of hexagonal cells (PHC)—were analyzed using a non-contact specular microscope (EM-3000; Topcon Corporation, Tokyo, Japan). The nuclear firmness was graded according to the Emery classification system [20]. Additionally, grades II and III were each subdivided into two additional levels, resulting in classifications of 2.0, 2.5, 3.0, and 3.5 for a more precise assessment of nuclear density. All biometric measurements were obtained using the optical biometer (OA-2000; Tomey, Nagoya, Japan). Axial length and ACD were measured using a swept-source optical coherence tomograph with a laser wavelength of 1060 nm [21].

### 2.3. New Surgical Instruments

To improve the precision and efficiency of the eight-chop technique, a new generation of surgical instruments has been developed [16,17]. The researchers conceptualized and designed a specialized set of choppers tailored for this technique and collaborated with a surgical instrument manufacturer to produce the devices. The Eight-chopper I (SP-8193; ASICO, Parsippany, NJ, USA) was specifically designed for grade II cataracts, featuring a reduced-diameter tip compared to conventional prechoppers. It measures 3.2 mm in length and 1.4 mm in width and incorporates a more pronounced leading edge to facilitate efficient nuclear division with minimal mechanical stress on the lens structure. For grade III cataracts, the Eight-chopper II (SP-8402; ASICO, Parsippany, NJ, USA) was developed with a smaller angular tip measuring 2.5 mm in length and 0.8 mm in width. Its design allows for vertical insertion into the lens nucleus, optimizing nuclear fragmentation while minimizing unnecessary intraocular manipulation. The incorporation of these specialized choppers into the eight-chop technique aims to improve surgical outcomes by reducing phacoemulsification energy requirements and preserving corneal endothelial integrity.

### 2.4. Surgical Technique

All phacoemulsification procedures with the Centurion^®^ phacoemulsification unit (Alcon Laboratories, Inc., Irvine, CA, USA) were performed by the same experienced surgeon who was proficient in the eight-chop technique. A clear, temporal corneal incision (3.0 mm) was created with a steel keratome in all cases. Sodium hyaluronate was injected into the anterior chamber. A continuous curvilinear capsulorhexis of 6.2–6.5 mm was created with capsule forceps. The soft-shell technique [22] was used for grade III cataracts.

A 27-gauge cannula was used for hydrodissection, but hydrodelineation was not performed. The lens nucleus was divided into eight segments using either Eight-chopper I or Eight-chopper II, depending on the nuclear density (grade II or grade III, respectively). An ophthalmic viscosurgical device (OVD) was injected into the anterior chamber, and the eight-chopper was inserted into the central lens nucleus, ensuring complete division. The nucleus was initially divided into two equal halves, after which it was rotated 90 degrees for further fragmentation. Subsequently, each heminucleus was bisected, and the nucleus was rotated 180 degrees, completing four divisions. The lens was then rotated 45 degrees, undergoing four additional divisions, yielding eight nuclear fragments. At the iris level, the eight nuclear fragments were phacoemulsified and aspirated. The capsular bag was completely cleared using the irrigation and aspiration tip, ensuring the complete removal of cortical material. A foldable IOL (Acrysof^®^ MN60AC, Alcon Laboratories, Inc., Fort Worth, TX, USA) with polymethylmethacrylate haptics was implanted into the capsular bag using an injector system, followed by OVD aspiration.

The phacoemulsification system settings included a maximum ultrasound power of 80%, an aspiration flow rate of 32 mL/min, and a 1.1 mm tip. If necessary, corneal stromal hydration was performed to seal the incision. At the end of the procedure, the anterior chamber was exchanged with a balanced salt solution containing moxifloxacin (0.5 mg/mL) to reduce postoperative infection risk.

### 2.5. Outcome Measures and Data Collection

The intraoperative outcome parameters consisted of operative time (minutes), phaco time (seconds), aspiration time (seconds), CDE, volume of fluid used (mL), and intraoperative complication rate. Operative time was counted from the beginning of the corneal incision to the end of the OVD aspiration. All surgical operations were imaged with a high-resolution surgical camera (MKC-704KHD, Ikegami, Tokyo, Japan), and the video images were captured and stored on a hard disk for documentation and subsequent analysis. Postoperative follow-up was conducted at postoperative days 1 and 2, weeks 1, 3, 7, and 19, and year 1. The postoperative outcome metrics included BCVA, IOP, CECD, CCT, CV, and PHC, which were evaluated at 7 weeks, 19 weeks, and 1 year postoperatively.

### 2.6. Statistical Analysis

An unpaired *t*-test was employed to compare intraoperative and postoperative outcomes between the SAC and control groups. Repeated two-way analysis of variance was used to analyze significant differences between the SAC and control groups and postoperative changes in BCVA, IOP, CECD, CCT, CV, and PHC. A chi-square test was conducted to assess differences in sex distribution between the SAC and control groups. A *p*-value of <0.05 was considered statistically significant for all analyses.

## 3. Results

This study included 160 eyes from 99 patients diagnosed with cataracts who underwent phacoemulsification and posterior chamber IOL implantation. The patient characteristics and intraoperative parameters are summarized in Table 1. No significant differences were observed in mean age or sex distribution between the SAC and control groups. However, ACD and axial length were significantly different between the two groups. There were no significant differences between the SAC and control groups in terms of operative time, phaco time, aspiration time, and CDE; however, lens hardness and the volume of fluid used were significantly different between the two groups.

Table 2 summarizes the preoperative and postoperative changes in BCVA. Postoperatively, both the SAC group and the control group showed significant improvements in BCVA. However, there were no significant differences between the SAC and control groups between the preoperative and 1-year postoperative periods.

Table 3 presents the preoperative and postoperative changes in CECD measurements. There was a significant difference in CECD between the SAC and control groups between the preoperative and 1-year postoperative periods. However, CECD did not significantly decrease in either group up to 1 year postoperatively.

Table 4 presents the preoperative and postoperative changes in CCT, CV, and PHC. There was a significant difference in CCT between the SAC and control groups between the preoperative and 1-year postoperative periods. However, there were no significant changes in CCT in either group up to 1 year postoperatively.

There was no significant difference in CV between the SAC and control groups between the preoperative and 1-year postoperative periods. However, there were significant changes in CV in both groups up to 1 year postoperatively. There was a significant difference in PHC between the SAC and control groups between the preoperative and 1-year postoperative periods. Furthermore, there were significant changes in PHC in both groups up to 1 year postoperatively.

Table 5 presents the changes in IOP. There was a significant difference in IOP between the SAC and control groups between the preoperative and 1-year postoperative periods. Furthermore, there were significant decreases in IOP in both groups up to 1 year postoperatively.

Table 6 presents the Pearson correlation coefficients between CECD loss at 1 year postoperatively and various preoperative and intraoperative parameters. The analysis revealed significant correlations between CECD loss and CDE, as well as a preoperative PHC in the total patient cohort. In the SAC group, significant correlations were observed between CECD loss and CDE, preoperative CCT, preoperative CV, and preoperative PHC. However, in the control group, no significant correlations were observed between CECD loss and any of the evaluated parameters.

In both the SAC and control groups, no intraoperative complications or capsulorhexis tears were detected.

## 4. Discussion

The parameters for the eight-chop technique of phacoemulsification surgery in the SAC group were as follows: 4.7 min for operative time, 15.4 s for phaco time, 65.6 s for aspiration time, 5.87 for CDE, and 26.6 mL for volume of fluid used, all of which represent extremely favorable values. Among these parameters, the only significant difference from the control group was observed in the volume of fluid used, which was 2.1 mL higher in the SAC group.

Postoperative BCVA recovery was similar between the SAC and control groups. Additionally, CECD loss in the SAC group was 1.3% at 7 weeks, 1.1% at 19 weeks, and 0.9% at 1 year postoperatively, which were similarly low compared to the control group. There was a significant difference in CECD between the SAC and control groups during the observation period. However, there was no significant decrease in CECD after surgery in either group, and SAC did not affect the loss of CECD. The reason for the significant difference in CECD during the observation period cannot be determined in this study, and further research is needed to elucidate this aspect.

No correlation was found between CECD loss and ACD in either the SAC or control group. However, when analyzing all cases, significant correlations were identified between CECD loss and CDE, as well as CECD loss and preoperative PHC. There was no correlation between CECD loss and ACD in the overall analysis. Regarding corneal endothelial cell parameters, there was a significant difference in CCT between the SAC and control groups during the observation period. However, there were no significant changes in CCT after surgery in either group. These findings suggest that differences in corneal endothelial cell function exist between the two groups. It is possible that the corneal endothelial cell function in the SAC group remains impaired for an extended period postoperatively, but further investigation is required to confirm this observation. Although significant changes in CV and PHC were observed in the SAC and control groups postoperatively, there was no significant difference in PHC between the two groups; on the other hand, there was a significant difference in CV between the two groups during the observation period. These results suggest that wound healing improves up to 1 year postoperatively, regardless of ACD. However, the natural course of events cannot be excluded, and further studies are needed to validate this hypothesis.

Cataract surgery is performed in a limited and narrow space. However, by ensuring sufficient surgical space, the risk of damage to the corneal endothelial cells can be significantly reduced. It is, therefore, crucial to provide adequate surgical space to minimize corneal endothelial cell damage during surgery. In cases with SAC depth, the surgical procedure must often be performed close to the corneal endothelium. This proximity increases the likelihood of CECD loss during cataract surgery [23]. Walkow et al. [23] identified shorter axial length and longer phaco time as significant risk factors for greater CECD loss. Additionally, ACD and axial length are reported to influence the corneal endothelial cells during phacoemulsification and are considered preoperative risk factors for CECD loss [18,24]. Eyes with SAC depth, particularly those with relatively dense cataracts, may be more vulnerable to CECD loss during phacoemulsification [25]. In this study, there were no significant CECD losses in both the SAC and control groups postoperatively. Additionally, no correlation was found between ACD and CECD loss. Previous studies have reported CECD losses of 9.36% [18] and 6.04% to 12.94% [25] at 8 weeks postoperatively. Similarly, in studies comparing femtosecond laser-assisted cataract surgery and conventional phacoemulsification, CECD losses of 5.85% and 8.23%, respectively, were observed at 24 weeks postoperatively [26]. In contrast, the present study demonstrated a CECD loss of 1.3% at 7 weeks postoperatively and 0.9% at 1 year postoperatively, representing approximately one-fourth of the results reported in the best previous studies. The significantly lower CECD loss observed in this study is strongly associated with the small volume of fluid used [27,28]; 8.0% to 8.23%, six times higher than that reported in this study, accompanied by fluid volumes of 55.4 mL to 67.25 mL [12,26], more than double the volume of fluid used in the eight-chop technique [12,26]. Differences in surgical techniques may also contribute to variations in CECD loss. Storr-Paulsen et al. [29] reported that the divide-and-conquer technique may result in greater CECD loss compared to the phaco-chop technique. It is plausible that differences in surgical techniques influence both the volume of fluid used and postoperative CECD loss. However, in this study, no significant correlation was found between the volume of fluid used and CECD loss. One possible explanation is that the volume of fluid used in the eight-chop technique is consistently low, potentially limiting the ability to detect correlations within this narrow range. Therefore, while the present results do not show a direct correlation, they do not rule out the possibility of a relationship between the volume of fluid used and CECD loss.

This study did not include a direct comparison with the divide-and-conquer technique or the phaco-chop technique. Therefore, it is necessary to interpret these findings in the context of studies that have evaluated different surgical techniques. A comparative analysis with these techniques would provide further insight into the efficacy and safety of the eight-chop technique, particularly regarding its impact on CECD loss and fluid usage.

Additionally, in this study, the ACD of the SAC group ranged from 2.33 mm to 3.00 mm. However, the impact of the eight-chop technique on cases with even shallower anterior chambers remains unclear. Further studies are required to assess whether the CECD loss, fluid dynamics, and surgical outcomes differ significantly in eyes with an ACD of less than 2.33 mm, where the space for phacoemulsification is even more restricted. Moreover, most of the cases included in this study had grade II nuclear hardness, and there applicability to grade III and IV nuclear hardness remains unverified. Cataracts with increased nuclear density may require higher phacoemulsification power, prolonged surgical time, and increased fluid usage, which could influence CECD loss and surgical efficiency. Further research is necessary to evaluate whether the eight-chop technique provides similar benefits in patients with more advanced nuclear sclerosis. The classification of ACD in this study was based on a division using the distance from the corneal epithelium to the anterior lens surface, with 3.00 mm as the threshold. This threshold was selected based on previous studies indicating that the average ACD is between 3.30 mm and 3.35 mm and does not typically decrease below 3.00 mm, even in individuals aged 80 years [19].

It was initially anticipated that SAC depth would reduce surgical efficiency, leading to a significant increase in CECD loss and intraoperative parameter values. However, in cataract surgery utilizing the eight-chop technique, there was no significant CECD loss in the SAC and control groups. Furthermore, the intraoperative parameters associated with the eight-chop technique demonstrated favorable values for the SAC group, indicating that this method maintains surgical efficiency despite the anatomical limitations posed by an SAC. Given these results, the eight-chop technique appears to be an effective and safe surgical approach, even in cases with SAC depth. Additionally, no correlation was found between CECD loss and ACD, suggesting that factors other than chamber depth, such as surgical technique and fluid dynamics, may play a more critical role in endothelial cell preservation during phacoemulsification.

Lens segmentation prior to phacoemulsification using the eight-chop technique may significantly reduce CECD loss by minimizing the volume of fluid used and enhancing the efficiency of lens nucleus removal. Similarly, femtosecond laser-assisted cataract surgery (FLACS) enables lens segmentation prior to phacoemulsification and has been proposed as a method to reduce CECD loss. Additionally, FLACS has the potential to decrease effective phaco time and CDE compared to conventional phacoemulsification, contributing to earlier visual recovery [30]. However, previous studies have demonstrated that the outcomes of FLACS are comparable to those of conventional phacoemulsification [30], with no significant advantage in terms of endothelial cell preservation. Notably, studies have reported that the eight-chop technique outperforms FLACS in minimizing CECD loss [12,26,31]. These findings suggest that the eight-chop technique may represent a significant advancement in phacoemulsification surgery, offering an alternative that optimizes ultrasound energy usage while preserving corneal endothelial cells. In other words, the eight-chop technique has the potential to emerge as a superior successor to conventional phacoemulsification surgery, particularly in addressing the limitations that FLACS sought to overcome.

Cataract surgery remains a leading cause of bullous keratopathy [5], and there is need for safe and efficient surgical techniques that minimize postoperative complications, particularly CECD loss. The eight-chop technique has demonstrated remarkable safety and efficiency, effectively inhibiting CECD loss postoperatively. Its widespread adoption has the potential to significantly improve surgical outcomes and contribute to better visual prognoses for patients with cataracts worldwide. Integrating this technique into standard cataract surgery protocols may enhance postoperative corneal health, reducing the risk of bullous keratopathy and other endothelial complications, thereby optimizing long-term visual function for patients undergoing cataract extraction.

## 5. Conclusions

This study found no correlation between ACD and CECD loss. In patients with SAC depth, CECD loss remained low (0.9%) at 1 year postoperatively following cataract surgery using the eight-chop technique. Additionally, key intraoperative parameters, including operative time, phaco time, aspiration time, CDE, and volume of fluid used, demonstrated favorable values, indicating high surgical efficiency and minimal endothelial trauma. Given these findings, the eight-chop technique is considered a minimally invasive, efficient, and endothelial-cell-preserving surgical approach for patients with SAC depth. Its adoption may contribute to enhanced corneal endothelial preservation and improved long-term surgical outcomes in patients with cataracts.

## Figures and Tables

**Table 1:** Preoperative characteristics and intraoperative parameters.

Characteristic/Parameter	SAC Group	Control Group	*p*-Value
Number of eyes	80	80	
Age (y)	74.9 ± 5.48	75.0 ± 6.2	0.11 ^a^
Gender: Men	22 (27.5%)	22 (27.5%)	1.0 ^b^
Women	58 (72.5%)	58 (72.5%)	
Anterior chamber depth (mm)	2.75 ± 0.19	3.32 ± 0.20	<0.01 ^c^
Axial length (mm)	22.93 ± 0.91	23.92 ± 1.29	<0.01 ^c^
Lens hardness	2.40 ± 0.30	2.26 ± 0.26	<0.01 ^c^
Operative time (min)	4.7 ± 1.1	4.5 ± 0.7	0.09 ^a^
Phaco time (s)	15.4 ± 6.1	13.9 ± 3.7	0.06 ^a^
Aspiration time (s)	65.6 ± 17.3	62.6 ± 11.3	0.19 ^a^
CDE	5.87 ± 2.01	5.59 ± 1.56	0.34 ^a^
Volume of fluid used (mL)	26.6 ± 8.1	24.5 ± 4.9	<0.05 ^c^

Unless otherwise specified, values are presented as mean ± standard deviation (SD) or percentages. ^a^: Unpaired *t*-test indicated no significant differences between the SAC and control groups. ^b^: Chi-square test indicated no significant differences between the SAC and control groups. ^c^: Unpaired *t*-test indicated significant differences between the SAC and control groups. CDE: cumulative dissipated energy; SAC: shallow anterior chamber.

**Table 2:** Pre- and postoperative best-corrected visual acuity values.

Group (*n* = 80 Each)	Preoperatively	7 Weeks Postoperatively	19 Weeks Postoperatively	1 Year Postoperatively	*p*-Value
SAC logMAR	0.062 ± 0.121	−0.050 ± 0.051	−0.046 ± 0.060	−0.045 ± 0.065	0.96 ^a^
Control logMAR	0.120 ± 0.187	−0.064 ± 0.039	−0.070 ± 0.025	−0.066 ± 0.030	
*p*-Value	<0.01 ^b^				

Values are expressed as mean ± standard deviation (SD). ^a^: No significant difference was found between the SAC and control groups (repeated two-way analysis of variance). ^b^: A significant difference was found (repeated two-way analysis of variance) in postoperative changes. logMAR: logarithmic minimum angle of resolution; SAC: shallow anterior chamber.

**Table 3:** Pre- and postoperative CECD values.

Group (*n* = 80 Each)	Preoperatively	7 Weeks Postoperatively	19 Weeks Postoperatively	1 Year Postoperatively	*p*-Value
SAC (cells/mm^2^) loss (%)	2678.3 ± 257.5	2640.5 ± 244.3 1.3 ± 2.1	2648.5 ± 247.6 1.1 ± 1.1	2653.2 ± 254.8 0.9 ± 1.8	<0.01 ^a^
Control (cells/mm^2^) loss (%)	2751.6 ± 254.7	2687.6 ± 248.7 2.3 ± 3.1	2730.8 ± 248.4 0.7 ± 2.3	2738.6 ± 252.9 0.5 ± 1.7	
*p*-Value	0.34 ^b^				

Values are expressed as mean ± standard deviation (SD). ^a^: A significant difference was found between the SAC and control groups (repeated two-way analysis of variance). ^b^: No significant differences were found in postoperative changes (repeated two-way analysis of variance). CECD: corneal endothelial cell density; SAC: shallow anterior chamber.

**Table 4:** Preoperative and postoperative changes in the CCT, CV, and PHC.

Group (*n* = 80 Each)	Preoperatively	7 Weeks Postoperatively	19 Weeks Postoperatively	1 Year Postoperatively	*p*-Value
	CCT (mean ± SD)				
SAC	514.9 ± 34.3	518.7 ± 35.6	518.9 ± 36.2	517.6 ± 35.4	<0.01 ^a^
Control	528.0 ± 31.9	533.4 ± 31.7	528.7 ± 32.1	527.1 ± 36.0	
*p*-Value	0.64 ^c^				
	CV (mean ± SD)				
SAC	39.6 ± 4.6	39.4 ± 4.0	39.0 ± 4.9	36.6 ± 3.6	0.63 ^b^
Control	38.9 ± 5.6	39.6 ± 5.4	38.5 ± 5.4	36.8 ± 5.6	
*p*-Value	<0.01 ^d^				
	PHC (mean ± SD)				
SAC	44.1 ± 6.9	44.4 ± 5.5	45.9 ± 5.5	47.2 ± 5.9	0.018 ^a^
Control	46.0 ± 6.0	44.8 ± 5.5	46.5 ± 5.6	48.8 ± 5.9	
*p*-Value	<0.01 ^d^				

Values are expressed as mean ± standard deviation (SD). ^a^: A significant difference was found between the SAC and control groups (repeated two-way analysis of variance). ^b^: No significant difference was found between the SAC and control groups (repeated two-way analysis of variance). ^c^: No significant changes were found in postoperative changes (repeated two-way analysis of variance) in the SAC and control groups. ^d^: Significant changes were found in postoperative changes (repeated two-way analysis of variance) in the SAC and control groups. CCT: central corneal thickness; CV: coefficient of variation; PHC: percentage of hexagonal cells; SD: standard deviation; SAC: shallow anterior chamber.

**Table 5:** Mean IOP and mean reduction of IOP in the course of time.

Group (*n* = 80 Each)	Preoperatively	7 Weeks Postoperatively	19 Weeks Postoperatively	1 Year Postoperatively	*p*-Value
SAC (mmHg) % decrease	13.7 ± 2.1	11.3 ± 1.9 16.7 ± 11.6	11.6 ± 2.0 14.9 ± 10.1	12.3 ± 1.9 9.7 ± 9.9	<0.01 ^a^
Control (mmHg) % decrease	13.2 ± 1.8	11.7 ± 1.6 10.9 ± 9.3	12.3 ± 1.6 6.6 ± 10.0	12.6 ± 1.7 3.8 ± 11.2	
*p*-Value	<0.01 ^b^				

Unless otherwise specified, values are expressed as mean ± standard deviation (SD) or percentages. ^a^: A significant difference was found (repeated two-way analysis of variance) between the SAC and control groups. ^b^: Significant differences were found (repeated two-way analysis of variance) in postoperative decreases in the SAC and control groups. IOP: intraocular pressure; SAC: shallow anterior chamber.

**Table 6:** Pearson’s correlation coefficients between CECD loss at 1 year postoperatively and various parameters.

	SAC Group (*n* = 80)		Control Group (*n* = 80)		Total (*n* = 160)	
Parameters	*r*-Value	*p*-Value	*r*-Value	*p*-Value	*r*-Value	*p*-Value
Age	0.062	0.587	−0.058	0.607	0.025	0.750
Lens hardness	0.083	0.466	0.035	0.759	0.094	0.237
Anterior chamber depth	−0.058	0.609	−0.105	0.356	−0.152	0.055
Axial length	0.023	0.837	0.058	0.610	−0.008	0.924
Operative time	−0.006	0.960	−0.029	0.801	0.003	0.966
Phaco time	0.133	0.240	−0.056	0.621	0.081	0.310
Aspiration time	0.048	0.672	−0.071	0.530	0.016	0.841
CDE	0.318	0.004 ^a^	−0.027	0.811	0.179	0.023 ^a^
Volume of fluid used	0.046	0.687	−0.087	0.441	0.017	0.827
Preoperative IOP	0.159	0.159	−0.016	0.885	0.025	0.750
Preoperative BCVA	0.169	0.135	−0.118	0.296	−0.025	0.755
Preoperative CECD	0.127	0.263	0.169	0.134	0.138	0.082
Preoperative CCT	0.240	0.032 ^a^	0.013	0.906	0.105	0.185
Preoperative CV	−0.226	0.044 ^a^	0.063	0.578	−0.062	0.433
Preoperative PHC	0.342	0.002 ^a^	0.066	0.560	0.195	0.014 ^a^

^a^: Statistically significant correlations. CECD: corneal endothelial cell density; SAC: shallow anterior chamber; CDE: cumulative dissipated energy; BCVA: best-corrected visual acuity; CV: coefficient of variation; PHC: percentage of hexagonal cells; CCT: central corneal thickness; IOP: intraocular pressure.

## Data Availability

The data presented in this study are available upon request from the corresponding author due to privacy and ethical restrictions.

## Abbreviations

The following abbreviations are used in this manuscript:

ACD	Anterior chamber depth
CECD	Corneal endothelial cell density
SAC	Shallow anterior chamber
BCVA	Best-corrected visual acuity
IOP	Intraocular pressure
CDE	Cumulative dissipated energy
IOL	Intraocular lens
CCT	Central corneal thickness
CV	Coefficient of variation
PHC	Percentage of hexagonal cells
OVD	Ophthalmic viscosurgical device
SD	Standard deviation

## References

[1] Forooghian F., Agrón E., Clemons T.E., Ferris F.L., Chew E.Y. (2009). Visual acuity outcomes after cataract surgery in patients with age-related macular degeneration: Age-related eye disease study report no. 27. Ophthalmology.

[2] Nderitu P., Ursell P. (2019). Iris hooks versus a pupil expansion ring: Operating times, complications, and visual acuity outcomes in small pupil cases. J. Cataract Refract. Surg..

[3] Chen H.C., Huang C.W., Yeh L.K., Hsiao F.C., Hsueh Y.J., Meir Y.J., Chen K.J., Cheng C.M., Wu W.C. (2021). Accelerated corneal endothelial cell loss after phacoemulsification in patients with mildly low endothelial cell density. J. Clin. Med..

[4] Waring G.O., Bourne W.M., Edelhauser H.F., Kenyon K.R. (1982). The corneal endothelium. Normal and pathologic structure and function. Ophthalmology.

[5] Feizi S. (2018). Corneal endothelial cell dysfunction: Etiologies and management. Ther. Adv. Ophthalmol..

[6] Bourne W.M. (1998). Clinical estimation of corneal endothelial pump function. Trans. Am. Ophthalmol. Soc..

[7] Kinoshita S., Koizumi N., Ueno M., Okumura N., Imai K., Tanaka H., Yamamoto Y., Nakamura T., Inatomi T., Bush J. (2018). Injection of Cultured Cells with a ROCK Inhibitor for Bullous Keratopathy. N. Engl. J. Med..

[8] Claesson M., Armitage W.J., Stenevi U. (2009). Corneal oedema after cataract surgery: Predisposing factors and corneal graft outcome. Acta Ophthalmol..

[9] Vasavada A., Singh R. (2000). Phacoemulsification in eyes with a small pupil. J. Cataract Refract. Surg..

[10] Igarashi T., Ohsawa I., Kobayashi M., Umemoto Y., Arima T., Suzuki H., Igarashi T., Otsuka T., Takahashi H. (2019). Effects of hydrogen in prevention of corneal endothelial damage during phacoemulsification: A prospective randomized clinical trial. Am. J. Ophthalmol..

[11] Park J., Yum H.R., Kim M.S., Harrison A.R., Kim E.C. (2013). Comparison of phaco-chop, divide-and-conquer, and stop-and-chop phaco techniques in microincision coaxial cataract surgery. J. Cataract Refract. Surg..

[12] Dzhaber D., Mustafa O., Alsaleh F., Mihailovic A., Daoud Y.J. (2020). Comparison of changes in corneal endothelial cell density and central corneal thickness between conventional and femtosecond laser-assisted cataract surgery: A randomised, controlled clinical trial. Br. J. Ophthalmol..

[13] Takahashi H. (2016). Corneal Endothelium and Phacoemulsification. Cornea.

[14] O’Brien P.D., Fitzpatrick P., Kilmartin D.J., Beatty S. (2004). Risk factors for endothelial cell loss after phacoemulsification surgery by a junior resident. J. Cataract Refract. Surg..

[15] Reuschel A., Bogatsch H., Oertel N., Wiedemann R. (2015). Influence of anterior chamber depth, anterior chamber volume, axial length, and lens density on postoperative endothelial cell loss. Graefe’s Arch. Clin. Exp. Ophthalmol..

[16] Sato T. (2023). Efficacy and safety of the eight-chop technique in phacoemulsification for patients with Cataract. J. Cataract Refract. Surg..

[17] Sato T. (2024). Eight-chop technique in phacoemulsification using iris hooks for patients with cataracts and small pupils. J. Clin. Med..

[18] Khalid M., Ameen S.S., Ayub N., Mehboob M.A. (2019). Effects of anterior chamber depth and axial length on corneal endothelial cell density after phacoemulsification. Pak. J. Med. Sci..

[19] Fernández-Vigo J.I., Fernández-Vigo J., Macarro-Merino A., Fernández-Pérez C., Martínez-de-la-Casa J.M., García-Feijoó J. (2016). Determinants of anterior chamber depth in a large Caucasian population and agreement between intra-ocular lens Master and Pentacam measurements of this variable. Acta Ophthalmol..

[20] Emery J.M., Emery J.M., Mclyntyre D.J. (1983). Kelman phacoemulsification; patient selection. Extracapsular Cataract Surgery.

[21] Huang J., Savini G., Hoffer K.J., Chen H., Lu W., Hu Q., Bao F., Wang Q. (2017). Repeatability and interobserver reproducibility of a new optical biometer based on swept-source optical coherence tomography and comparison with IOLMaster. Br. J. Ophthalmol..

[22] Miyata K., Nagamoto T., Maruoka S., Tanabe T., Nakahara M., Amano S. (2002). Efficacy and safety of the soft-shell technique in cases with a hard lens nucleus. J. Cataract Refract. Surg..

[23] Walkow T., Anders N., Klebe S. (2000). Endothelial cell loss after phacoemulsification: Relation to preoperative and intraoperative parameters. J. Cataract Refract. Surg..

[24] Lee N.S., Ong K. (2024). Risk factors for corneal endothelial cell loss after phacoemulsification. Taiwan J. Ophthalmol..

[25] Hwang H.B., Lyu B., Yim H.B., Lee N.Y. (2015). Endothelial cell loss after phacoemulsification according to different anterior chamber depths. J. Ophthalmol..

[26] Mencucci R., De Vitto C., Cennamo M., Vignapiano R., Buzzi M., Favuzza E. (2020). Femtosecond laser-assisted cataract surgery in eyes with shallow anterior chamber depth: Comparison with conventional phacoemulsification. J. Cataract Refract. Surg..

[27] Hayashi K., Nakao F., Hayashi F. (1994). Corneal endothelial cell loss after phacoemulsification using nuclear cracking procedures. J. Cataract Refract. Surg..

[28] Kohlhaas M., Klemm M., Kammann J., Richard G. (1998). Endothelial cell loss secondary to two different phacoemulsification techniques. Ophthalmic Surg. Lasers.

[29] Storr-Paulsen A., Norregaard J.C., Ahmed S., Storr-Paulsen T., Pedersen T.H. (2008). Endothelial cell damage after cataract surgery: Divide-and-conquer versus phaco-chop technique. J. Cataract Refract. Surg..

[30] Roberts T.V., Lawless M., Bali S.J., Hodge C., Sutton G. (2013). Surgical outcomes and safety of femtosecond laser cataract surgery: A prospective study of 1500 consecutive cases. Ophthalmology.

[31] Cruz J.C.G., Moreno C.B., Soares P., Moscovici B.K., Colombo-Barboza G.N., Colombo-Barboza L.R., Colombo-Barboza M.N. (2023). Comparison of endothelial cell loss in diabetic patients after conventional phacoemulsification and femtosecond laser-assisted cataract surgery. BMC Ophthalmol..

